# Highway Visibility Estimation in Foggy Weather via Multi-Scale Fusion Network

**DOI:** 10.3390/s23249739

**Published:** 2023-12-10

**Authors:** Pengfei Xiao, Zhendong Zhang, Xiaochun Luo, Jiaqing Sun, Xuecheng Zhou, Xixi Yang, Liang Huang

**Affiliations:** 1 Key Laboratory of Transportation Meteorology, China Meteorological Administration, Nanjing 210019, China; nuist_xiaopengfei@163.com (P.X.); 13851612368@163.com (Z.Z.); jqsun115@163.com (J.S.); zhouxuechengnju@163.com (X.Z.); yangqq2014@lzu.edu.cn (X.Y.); 2 Jiangsu Provincial Meteorological Service Center, Nanjing 210019, China

**Keywords:** visibility estimation, image classification, multi-scale fusion network

## Abstract

Poor visibility has a significant impact on road safety and can even lead to traffic accidents. The traditional means of visibility monitoring no longer meet the current needs in terms of temporal and spatial accuracy. In this work, we propose a novel deep network architecture for estimating the visibility directly from highway surveillance images. Specifically, we employ several image feature extraction methods to extract detailed structural, spectral, and scene depth features from the images. Next, we design a multi-scale fusion network to adaptively extract and fuse vital features for the purpose of estimating visibility. Furthermore, we create a real-scene dataset for model learning and performance evaluation. Our experiments demonstrate the superiority of our proposed method to the existing methods.

## 1. Introduction

Atmospheric visibility is a measure of how transparent the atmosphere is. For traffic safety, atmospheric visibility monitoring is one of the essential services [1]. Atmospheric visibility can be measured by sensors or visual perception. These sensors include meteorological sensors and forward scatter visibility sensors. However, the meteorological observation network is not dense enough to monitor agglomerate fog. Additionally, visibility meters are costly and inaccurate in non-uniform atmospheric conditions [2].

Another method for estimating visibility is based on visual perception. This method processes real-time images captured through surveillance to estimate visibility. Methods based on visual perception can be divided into three main types: multi-image-based methods, Koschmieder-based methods, and deep-learning-based methods.

The multi-image-based methods require multiple images to establish a relationship between image and visibility. Some researchers use the filtering methods (like the Sobel filter or homomorphic filter) to establish the relationship between image features and visibility [3,4]. However, these methods are sensitive to illumination variations. To overcome this disadvantage, Babari et al. [5] adopted the Lambertianess of the image as the reference to adjust the image contrast. Varjo and Hannuksela [6] proposed a new method based on feature vectors that were projections of the scene images with lighting normalization. Then, the new method was combined with the high-dynamic-range imaging to improve nighttime image quality. The above methods require ground truth data from sensors as a reference, so they are not suitable for real-time visibility estimation. To solve this problem, some methods based on Koschmieder’s law are proposed [7]. Koschmieder’s law describes the relationship between scene visibility and the extinction coefficient. Hautière et al. have conducted a series of studies along these lines [8,9,10,11,12]. These methods treat the distance between the camera and the furthest visible object as the scene visibility. The scene visibility can be obtained by calculating the Koschmieder’s law from the geometric calibration model of the camera. Negru and Nedevschi [13] estimated the scene visibility by detecting the positions of the inflection point and the horizontal line in the image. These methods can estimate the scene visibility without ground truth data from sensors. Nevertheless, the precision of geometric calibration is critical to these approaches.

The development of image recognition and deep learning technology has led to their gradual application in atmospheric visibility estimation. Deep-learning-based methods are superior for complex interaction processing compared to the above two methods. You et al. [14] combined CNNs (convolutional neural networks) and RNNs (recurrent neural networks) to estimate the relative atmospheric visibility from images, which significantly improved the estimation accuracy. However, the evaluation capacity of this model was only 300–800 m. Palvanov and Cho [15] proposed a deeply integrated convolutional neural network with three streams for visibility estimation. And this approach considered different image features as inputs to the model. Unlike the previous method using only visible light images, Wang et al. [16] used visible–infrared image pairs as the input and proposed a multimodal deep fusion model to learn the joint features from the input. Although the performance of the deep-learning-based methods was superior to the two methods mentioned above, the following problems still exist: (1) Most existing methods can not sufficiently extract fog features, which can provide valuable information for subsequent model learning; and (2) most existing methods are inadequate for extracting significant high-level multi-scale features for visibility estimation. Therefore, we propose a novel multi-scale fusion network for visibility estimation (Vis-MFN) from a single image. The main contributions are as follows:

(1) We propose a CNN-based method for highway visibility estimation from a single surveillance image. This method can provide low-cost and efficient support for intelligent highway management.
(2) A multi-scale fusion network model is developed to estimate visibility from the input highway surveillance image. We are more concerned with the efficient transfer of low-level features to high-level features than with the design of complex network structures. Multiple image feature extraction methods are utilized to extract low-level visual features of fog, which can provide valuable information for subsequent model learning. The multi-scale fusion module is designed to extract the important high-level multi-scale features for the final visibility estimation, which can effectively improve the accuracy of the estimation.
(3) We create a dataset of real-world highway surveillance images for model learning and performance evaluation. Each image in the dataset was labeled by professional traffic meteorology practitioners.

## 2. Proposed Method

**Figure 1:**
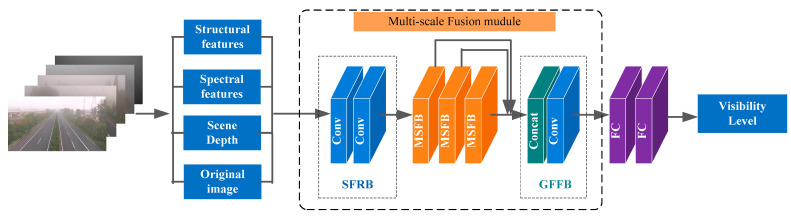
The network structure of Vis-MFN.

We designed a deep multi-scale fusion network for highway visibility estimation. As we all know, extracting visibility features from a single image is difficult. To better understand the scene in the image, we extracted different types of features from the input image to provide critical information for the subsequent model learning. The multi-scale fusion module was further designed to jointly learn visibility from these image features of the same image. The overall architecture of the proposed network is shown in Figure 1. Multiple algorithms were adopted to process the input image to obtain detailed structural, spectral, and scene depth features. The multi-scale fusion module was further designed to fuse these features for adaptive visibility estimation.

### 2.1. Image Feature Extraction

#### 2.1.1. Detailed Structural Feature Extraction

**Figure 2:**
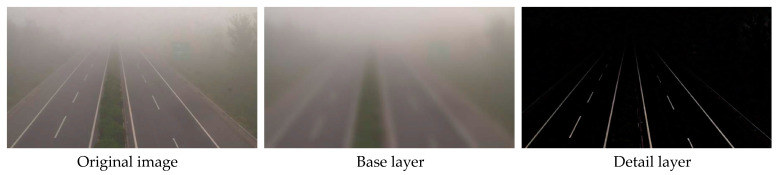
The original image, base layer, and detail layer. The detail layer is the detailed structural feature which we want.

This branch is designed to extract the detailed structural features. The presence of fog can obscure detailed structural features in the image. Therefore, the richness of such features can reflect the visibility to some extent. We adopt the fast guided filter in [17] to decompose the image into a base layer and a detail layer. The detail layer is the specific structural feature that we desire. The guided filter is a technique for edge-aware image filtering. We choose the fast guided filter because of its superior visual output, fast processing speed, and ease of implementation. The guidance image, filtering input image, and filtering output image are represented as I, p, and q, respectively. The guided filter can be denoted as follows:

(1) $$q_{i}=a_{k}I_{i}+b_{k},\;\forall i\in \omega_{k}$$

(2) $$a_{k}=\frac{\frac{1}{\left|\omega \right|}\sum_{i\in \omega_{k}}I_{i}p_{i}-\mu_{k}{\bar{p}}_{k}}{\sigma_{k}^{2}+\varepsilon }$$

(3) $$b_{k}={\bar{p}}_{k}-a_{k}\mu_{k}$$

where i is the index of a pixel and k is the index of a local square window ω with a radius r. $\mu_{k}$, and $\sigma_{k}$ are the mean and variance of I in the window k, and ε is a regularization parameter controlling the degree of smoothness. The filtering output is represented as follows:

(4) $$q_{i}={\bar{a}}_{i}I_{i}+{\bar{b}}_{i}$$

where ${\bar{a}}_{i}$ and ${\bar{b}}_{i}$ are the average of a and b, respectively, on the window $\omega_{i}$ at i. As shown in Figure 2, the detail layer mainly contains the detailed structural features, such as lane lines. Therefore, the fast guided filter is suitable for extracting detailed structural features of highway scene images.

#### 2.1.2. Spectral Feature Extraction

**Figure 3:**
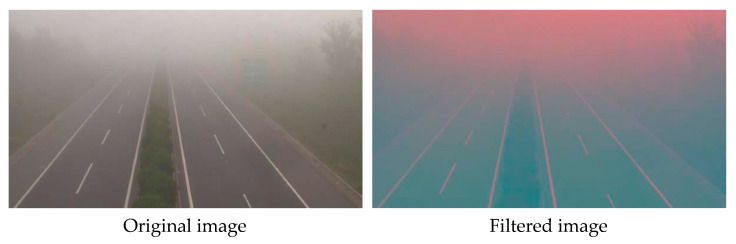
The original image and the filtered image.

This branch was designed to extract the areas covered by fog. Images captured in dense fog generally have low contrast, resulting in indistinct visuals. Because of the low contrast, fog images closely resemble grayscale images. To improve the visual characteristics of fog, we used a spectral filter, as the human visual system is more responsive to colors than grayscale. To facilitate the extraction of the spectral features of fog, we converted the images from RGB color space to LAB color space, since it covers the full range of human color perception. As shown in Figure 3, the regions of fog in the filtered image are marked in pink. The filtered image presents fog regions more prominently than the original image. As a result, the following CNN-based model can easily identify the fog regions and focus on extracting local features.

#### 2.1.3. Scene Depth Feature Extraction

**Figure 4:**
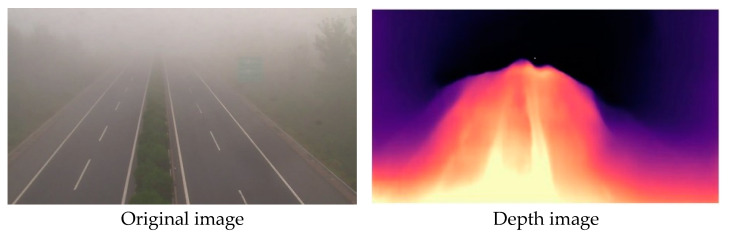
Depth feature extraction.

This branch is designed to extract the scene depth feature. The scene depth provides information about the depth of objects in the image, which is useful for visibility estimation. We utilized MiniNet [18] to extract the scene depth feature, as it is a lightweight and efficient network for unsupervised monocular depth prediction. As shown in Figure 4, the black area in the depth image is basically consistent with the fog area in the original image.

### 2.2. Multi-Scale Fusion Module

The multi-scale fusion module is designed to adaptively fuse the detailed structural, spectral, and scene depth features. It consists of three parts, including a shallow feature representation block (SFRB), multiple multi-scale fusion blocks (MSFBs), and a global feature fusion block (GFB). Specifically, two 3 × 3 convolutional layers extracted shallow features from the structural, spectral, and scene depth features. This process is defined as follows:

(5) $$S=f_{s}([X_{1},X_{2},X_{3},X_{4}])$$

where $f_{s}$ denotes the SFRB function; $X_{1},X_{2},X_{3}$ represent the structural, spectral, scene depth features, respectively; $X_{4}$ represents the original image; and [⋅] represents the concatenation operation. Then, several multi-scale fusion blocks (MSFBs) were designed to extract multi-scale features. This process can be defined as follows:

(6) $$M_{k}=f_{m}(M_{k-1}),\;k=1,\ldots ,n$$

where $f_{m}$ denotes the *m*-th MSFB function and $M_{k}$ and $M_{k-1}$ represent the input and output of the *k*-th MSFB, respectively. We fused these multi-scale features using the concatenation operation and further extracted high-level features through the convolution operation. This procedure can be formulated as follows:

(7) $$G=f_{g}([M_{1},M_{2},\ldots M_{n}])$$

where $\left[M_{1},M_{2},\ldots M_{n}\right]$ denotes the concatenation of the feature maps produced in each MSFN and $f_{g}$ represents the following convolution operation.

**Figure 5:**
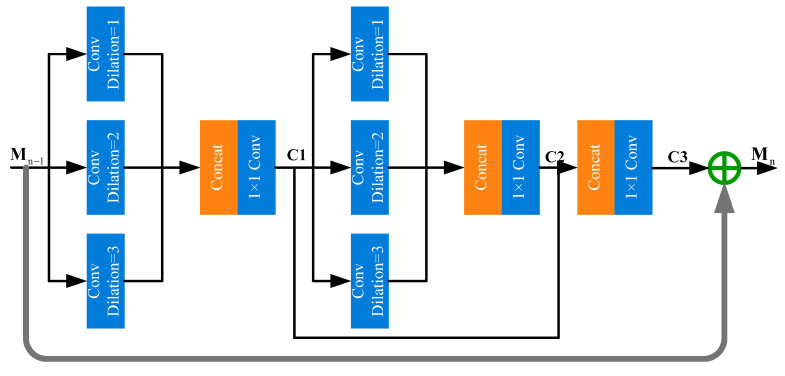
The architecture of the multi-scale fusion block.

The architecture of the multi-scale fusion block is shown in Figure 5. We constructed a three-bypass network using different convolutional kernels in each bypass for multi-scale feature extraction. We used multiple 3 × 3 dilated convolutions with varying dilation factors instead of convolutional kernels of various sizes to extract multi-scale features, which enlarged the receptive field and maintained the filter size [19]. We concatenated the features extracted by the dilated convolutions for multi-scale feature fusion and then used a 1 × 1 convolutional layer to reduce the dimension. Then, we concatenated the outputs of two 1 × 1 convolutional layers and used a 1 × 1 convolutional layer to reduce the dimension. This part can be denoted as follows:

(8) $$C_{1}=f_{c1}([f_{d1}(M_{n-1}),f_{d2}(M_{n-1}),f_{d3}(M_{n-1})])$$

(9) $$C_{2}=f_{c1}([f_{d1}(C_{1}),f_{d2}(C_{1}),f_{d3}(C_{1})])$$

(10) $$C_{3}=f_{c1}([C_{1},C_{2}])$$

where $f_{di}$ represents the dilated convolution with dilation factor i, and $f_{c1}$ represents 1 × 1 convolution. Finally, the residual structure was adopted to increase information flow, which helped to reduce the computational complexity and improved the performance of the model. The output of *k*-th MSFB can be expressed as follows:

(11) $$M_{n}=M_{n-1}+C_{3}$$

## 3. Experiments

### 3.1. Dataset

**Figure 6:**
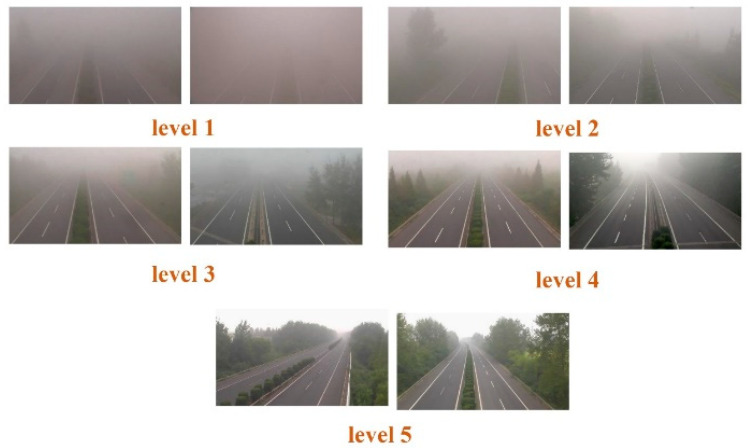
Some sample images in our dataset.

To our knowledge, there is no publicly available dataset of actual highway fog scenes. For this reason, we created a real-scene dataset for highway visibility estimation. Specifically, we selected about 30 cameras located near traffic weather stations in the Jiangsu section of the Beijing-Shanghai Expressway. These cameras were positioned more than 10 km from each other to guarantee diverse shooting scenarios. Then, we collected more than 18,000 surveillance images of real highway scenes obtained from these cameras. These images were collected in different time periods, further ensuring the diversity of the dataset. After similarity elimination and quality control, 15,000 images were selected as the training dataset and 3000 images were chosen as the test dataset. Meteorology professionals classified the fog intensity into five levels according to visibility. The detailed visibility level standard is shown in Table 1. All dataset images were automatically labeled based on observation data from traffic weather stations and subsequently adjusted by skilled traffic meteorology practitioners. Some sample images from the dataset are presented in Figure 6. The dataset is currently not accessible for public use due to security concerns regarding road data.

### 3.2. Implementation and Training Details 

We used Adam [20] for optimization. The momentum and weight decay were set to 0.9 and 0.0001, respectively. The learning rate was initialized to 0.0001 and decreased by a factor of 10 every 40 epochs. We implemented our models with Pytorch1.5.1 on a single GPU of NVIDIA RTX 3090. Cross-entropy was adopted as the loss function.

### 3.3. Comparison Experiments

We compared the proposed method with several deep-learning-based methods, including two image classification methods (AlexNet [21] and VGG16 [22]) and two atmospheric visibility estimation methods (relative CNN-RNN [14] and STCN-Net [23]). We re-trained these three deep-learning-based methods on our dataset, where the parameters were set according to the recommendations in the paper.

**Figure 7:**
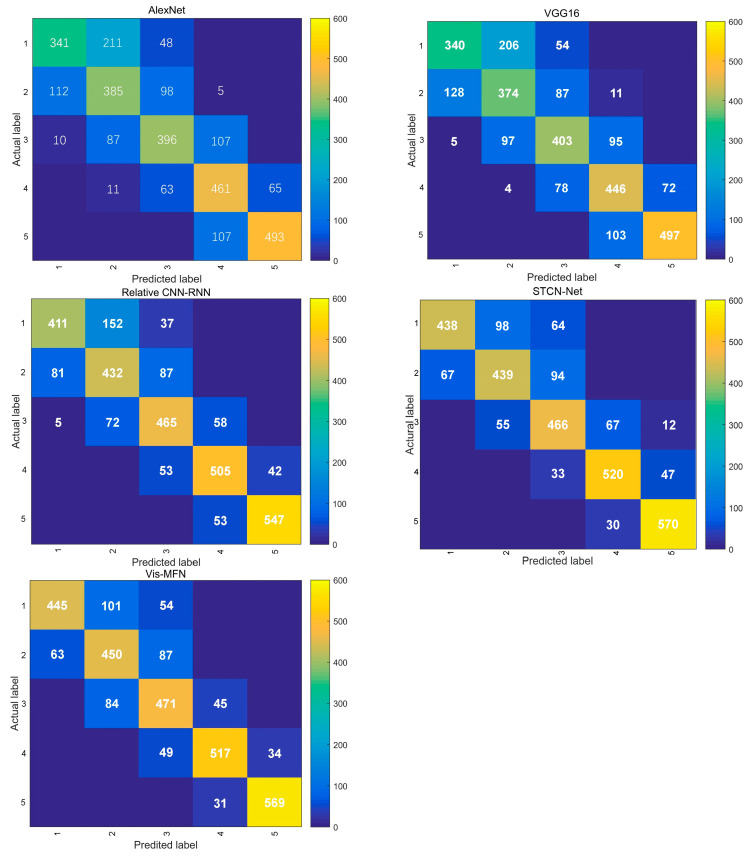
The confusion matrices of multiple methods on the test dataset.

Table 2 shows the accuracy of the multiple methods tested on the test dataset. The experimental results indicate that the AlexNet and VGG16 methods showed poor performance, primarily because the AlexNet and VGG16 models are designed for natural image classification and do not take into account the characteristics unique to road scene images. Consequently, extracting visibility features effectively is quite challenging. The relative CNN-RNN method outperformed AlexNet and VGG16 because the CNN-RNN module was able to capture the global view while approximating human attention shift, which enabled it to learn more effective visual features compared to AlexNet and VGG16. However, the relative CNN-RNN estimated visibility from only the original image, so it was challenging to extract multi-scale features. The STCN-Net achieved a better performance than the relative CNN-RNN method, since this method designed a novel 3D multi-feature stream matrix, which provided rich low-level features. However, the STCN-Net performed slightly less well in terms of visibility below 500 m. The proposed Vis-MFN achieved the best estimation accuracy. Figure 7 shows the confusion matrices of multiple methods on the test dataset. It can be seen that the classification labels of the proposed method are mainly clustered along the main diagonal of the matrix, demonstrating the stability of the classification performance.

**Figure 8:**
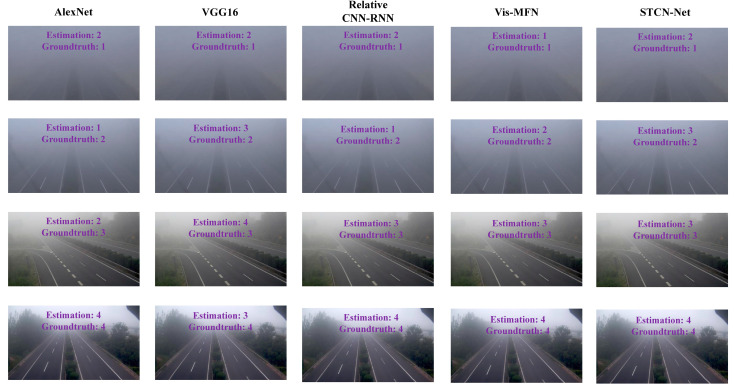
Visualization of the estimated results of different methods on images with different visibility levels. The estimated results and ground truth are marked on the image.

Figure 8 shows the estimated results of different methods on test images with different visibility levels. The proposed method was able to make correct estimates on sample images with different visibility levels, since the proposed method had two main advantages. The first advantage was that three image feature extraction algorithms could adequately extract visibility-related features from the images. The second advantage was that the multi-scale fusion module could adaptively extract useful features for visibility estimation.

### 3.4. Ablation Experiments

We further conducted ablation experiments to verify the effectiveness of the image feature extraction algorithms and the multi-scale fusion module.

(1) Vis-MFN-NF: No image feature extraction algorithm was used in the model.
(2) Vis-MFN-NM: The multi-scale fusion blocks were replaced by multiple convolutions in series. Meanwhile, the receptive field of the new network remained unchanged.
(3) Vis-MFN-M2: Only two multi-scale blocks were used in the network.
(4) Vis-MFN-M4: Four multi-scale blocks were used in the network.

Table 3 shows the estimation accuracy of the proposed method and two ablation methods. It can be seen that both the multi-scale fusion module and the image feature extraction methods significantly improved the estimation accuracy. Although the performance of Vis-MFN-M4 was better than that of Vis-MFN-M2, the complexity of Vis-MFN-M4 was significantly increased. Therefore, we chose the Vis-MFN-M2 for operational application.

## 4. Conclusions

Highway visibility estimation in foggy weather is important for traffic safety and intelligent highway management. Traditional methods utilize visibility sensors to directly measure visibility. However, the meteorological observation network is not dense enough to monitor agglomerate fog. Additionally, visibility sensors are costly and inaccurate in non-uniform atmospheric conditions. Although deep learning methods have been applied by many scholars for visibility estimation, the following problems still exist: (1) Most existing methods cannot sufficiently extract fog features, which can provide valuable information for subsequent model learning. (2) Most existing methods are inadequate in extracting significant, high-level, multi-scale features for visibility estimation. To close these research gaps, we propose a multi-scale fusion network for estimating highway visibility in foggy weather. The network can estimate the visibility level from a single image of a highway scene. To achieve this, we used multiple image feature extraction methods to extract the structural, spectral, and scene depth features from the image. Then, a multi-scale fusion module was designed to extract and fuse relevant features to estimate visibility adaptively. The experiments demonstrate the effectiveness of the proposed method. Our future work aims to improve the performance of our method and propose a benchmark for highway visibility estimation.

## Figures and Tables

**Table 1:** Visibility level standard.

Visibility Level	1	2	3	4	5
Visibility distance	0–50 m	50–100 m	100–200 m	200–500 m	500+ m

**Table 2:** The estimation accuracy of multiple methods on the test dataset. The best result is marked in bold.

	AlexNet	VGG16	Relative CNN-RNN	STCN-Net	Vis-MFN
Accuracy	69.21%	68.72%	78.58%	81.10%	**81.76%**

**Table 3:** The results of the ablation experiment. The best result is marked in bold.

Setting	Vis-MFN-NM	Vis-MFN-NF	Vis-MFN-M2	Vis-MFN-M4
Image feature extraction methods	✓	×	✓	✓
Multi-scale fusion module	×	✓	✓	✓
The number of MSFBs	2	2	2	4
Accuracy	75.36%	72.85%	81.76%	**82.49%**

## Data Availability

The corresponding author can provide data supporting the findings of this study upon request. Due to ethical and privacy concerns, the data are not publicly available.
